# Serious Games for Seniors With Dementia: A Pilot Study

**DOI:** 10.1177/07334648251350846

**Published:** 2025-06-12

**Authors:** Alexander Prinz, Katja Orlowski, Eberhard Beck, Kerstin Witte

**Affiliations:** 1 Department of Sports Engineering/Movement Science, 9376 Otto-von-Guericke-University Magdeburg, Magdeburg, Germany; 2 Department of Computer Science and Media, 38964 Brandenburg University of Applied Sciences, Brandenburg, Germany

**Keywords:** serious games, dementia, physical, cognitive training, balance training

## Abstract

Dementia, marked by cognitive decline, significantly impacts daily life. With global prevalence rising, traditional treatments manage symptoms but have side effects and offer no cure. Non-pharmacological interventions, like serious games, are gaining importance. This study assesses the feasibility and benefits of serious games for people with mild to moderate dementia over a 10-week intervention. Sixty-one patients were recruited, with 35 completing the study. The intervention included six games focusing on physical and cognitive training. Outcome measures were motor function, cognitive assessments, quality of life, and depression. Results showed significant improvements in dynamic balance (*p* = .013) but no significant changes in other measures. The findings suggest that serious games are feasible and can improve motor functions like balance. However, short intervention periods may limit their impact on cognitive function and quality of life. Longer interventions and personalized game designs are recommended for greater benefits.

**What this paper adds**

• The study was able to show that serious games can improve motor skills, especially dynamic balance, despite a short intervention period and that no significant deterioration occurred over this period.
• The paper identifies challenges and recommends that future research requires longer intervention periods and personalized game designs to achieve greater cognitive and quality-of-life improvements.

**Applications of study findings**

• Serious games can be a non-pharmacological alternative that, besides potential effects, brings variety and enjoyment to the daily lives of people with dementia.
• There is a need for standardized protocols and detailed guidelines for designing and implementing serious games to ensure consistent and reliable research outcomes.

## Introduction

Dementia is a complex and multifaceted condition characterized by a progressive decline in cognitive abilities, such as memory, reasoning, and judgment, which severely impacts daily functioning and independence. Dementia not only imposes a significant burden on individuals and their families but also presents an escalating challenge to global healthcare systems. It is estimated that by 2050, approximately 152 million individuals will be living with dementia worldwide (Long, S., Benoist, C., Weidner, W., 2023). Current treatment approaches for dementia are predominantly pharmacological, aiming to manage symptoms and slow disease progression. However, these medications often come with significant side effects, including nausea, dizziness, and an increased risk of falls, which can exacerbate the overall condition of older adults (Birks & Harvey, 2018). Consequently, there is a growing interest in non-pharmacological interventions that aim to enhance patients’ quality of life without adverse effects.

Non-pharmacological therapies have shown promising potential in addressing the multifaceted needs of people with dementia (PWD). These interventions encompass a variety of approaches, such as cognitive therapy, social interaction, and physical exercise programs. Among these, physical exercise has been particularly effective in improving motor skills, cognitive function, and emotional well-being (Heyn et al., 2004; Rolland et al., 2007). For instance, Heyn et al. (2004) conducted a meta-analysis that demonstrated significant improvements in physical and cognitive functions among PWD who participated in exercise programs. Similarly, Rolland et al. (2007) highlighted the benefits of physical activity on mobility and activities of daily living, which contribute to a better quality of life. Despite their efficacy, these programs are often resource-intensive, requiring trained personnel and individualized approaches to meet the specific needs of patients, particularly in group settings. This has limited their scalability and widespread adoption. Advances in technology, however, offer innovative solutions to overcome these challenges. Tools such as virtual reality (VR), Augmented Reality (AR), mobile applications, and serious games have emerged as accessible and engaging options to support therapeutic interventions tailored to the needs of PWD (Ipakchian Askari et al., 2024; Neal et al., 2021).

Serious games, in particular, stand out as a promising avenue for addressing the cognitive and physical challenges associated with dementia. These games, often referred to as exergames when they combine physical activity with gaming elements, are designed to provide therapeutic benefits while simultaneously engaging patients in enjoyable and adaptive experiences (Wiemeyer & Kliem, 2012). Research has demonstrated their potential to enhance motor skills, cognitive functions, and overall quality of life. For example, Abd-Alrazaq et al. (2022) found that serious games **may** improve memory and cognitive retention in older adults, while Anguera et al. (2013) showed that video games targeting cognitive training improved multitasking abilities. Ning et al. (2020) emphasized the growing role of serious games as a viable therapeutic tool for dementia, highlighting their adaptability to individual needs and their capacity to maintain engagement through personalized feedback and adjustable difficulty levels. A systematic review by Dietlein et al. (2018) synthesized findings on the feasibility and effects of serious games for PWD, offering valuable recommendations for future research. Among the key studies reviewed, one by Tárraga et al. (2006) demonstrated significant cognitive improvements in patients using an interactive multimedia cognitive stimulation tool, while another by Padala et al. (2012) highlighted the potential of serious games in improving balance and gait in elderly patients with dementia. These studies underscore the potential of serious games to address both physical and cognitive impairments comprehensively. The review identified substantial gaps, including the lack of standardized methodologies and long-term studies, which challenge the generalization of findings and the development of broader implementation strategies. Despite encouraging results, the research landscape remains fragmented, with significant heterogeneity in study designs, sample sizes, and outcome measures. This highlights the urgent need for more rigorous and systematic studies to assess the feasibility, effectiveness, and long-term impact of serious games on PWD.

Therefore, this pilot study aims to address these research gaps by exploring the feasibility of specially developed serious games for PWD. The main aim of this project was to investigate whether serious games can be used and played by people with mild to moderate dementia. Additionally, the study seeks to investigate whether these interventions can positively impact motor skills, cognitive function, quality of life, and levels of depression among participants. By addressing these critical questions, this research contributes to advancing our understanding of the potential role of serious games in improving the lives of PWD.

## Methods and Materials

### Study Design

**Figure 1.**
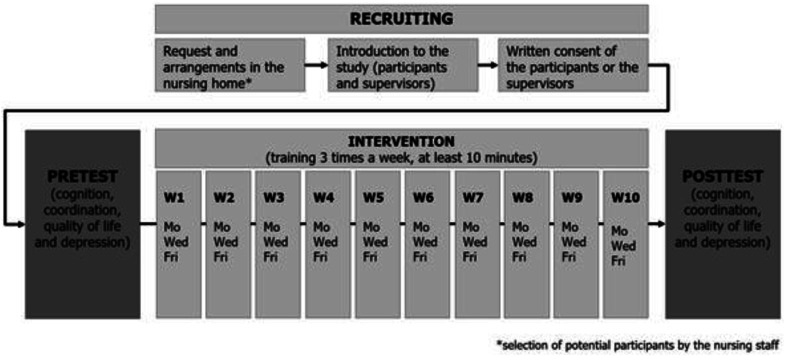
Overview of study design.

The present pilot study was designed as a 10-week intervention study with one intervention group, three times a week, 10 minutes per unit and two measurement time points (pre- and post-test, see details in Figure 1). The 10-week timeline was primarily determined by logistical and feasibility considerations (several facilities at different locations involved). The session duration of at least 10–15 minutes was chosen as this study was a pilot designed to assess feasibility and determine a manageable length, prioritizing engagement and balance without causing fatigue. The interventions were carried out by research assistants and supported by the institution’s staff. The participants were given appropriate guidance and support while playing.

**Figure 2.**
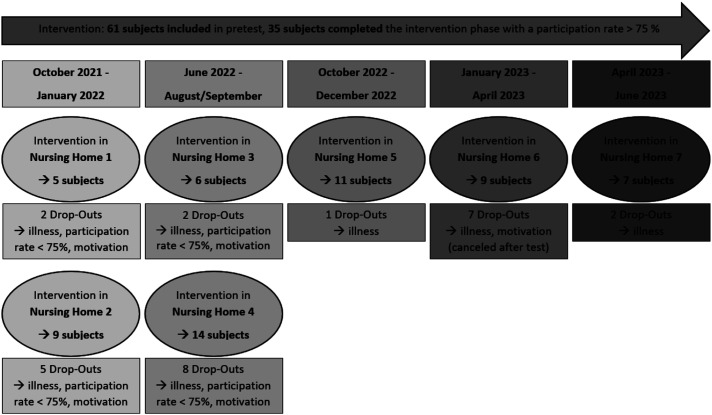
Timeline of the individual interventions in the seven nursing homes from October 2021 to June 2023.

The study was fully approved by the Ethics Committee of the authors’ institution and conducted by the Declaration of Helsinki. Additionally, it was registered in the German Clinical Trials Register. Data collection was conducted successively between October 2021 and June 2023 in seven nursing homes to obtain a sufficiently large sample size (see Figure 2). Written informed consent was obtained in advance from participants' legal representatives, and participants themselves were thoroughly informed about the study’s purpose during the first meeting.

### Sample Description

Potential subjects were searched in seven nursing homes from September 2021 to April 2023. The nursing staff recruited subjects from the facilities because they knew the potential participants better. For this purpose, the relatives or legal representatives of the potential subjects were contacted and informed about the study in consultation with the cooperating institutions. An informed consent form obtained their consent. However, some legal representatives did not want their relatives to participate in an exercise program. The nursing staff was aware of this problem and was, therefore, able to make a preliminary selection. The following inclusion criteria guided the nursing staff: Subjects were included when their Mini-Mental State Examination (MMSE) value was larger than 10, PWD had to be able to stand unaided, without support or assistance, for a short time (at least 1 minute) and to walk alone a short walkway (walking aid, such as walker or crutches were permitted). Exclusion criteria were hypertension, severe cardiovascular diseases such as cardiac arrhythmias, renal insufficiency, and severe motor impairment (inability to independently perform basic motor tasks such as standing unaided for a short time, maintaining postural stability for a short period or use a wheelchair) and completed less than 75% units. Sixty-one PWD were included in the study in the period and settings described (48 female/13 male; age: 83.02 ± 6.68 years).

### Serious Games

The superordinate name (collection) of all developed games is KoKoFIT. KoKoFIT is a German abbreviation that includes the three words “Kognition,” “Koordination,” and “Fitness,” which means that cognitive and physical functions/abilities are trained to age healthily. The development of the KoKoFIT games series started in 2018 and was performed with a user-centered design. KoKoFIT is a collection of six serious games with a physical or a cognitive focus. The KoKoFIT games were designed to be controlled by placing the body’s center of gravity (COG) on a force plate. This allows for posture and mobility to be trained and coordination to be increased. The games require balance skills to overcome obstacles or achieve goals. For developing serious games for PWD, focus group discussions were held with various local older adult advisory council representatives. These discussions aimed to gain an initial understanding of the needs, requirements, and preferences of the target group, which led to the development of the games (see Table 1). During the initial interventions, it was observed that physically and cognitively fitter PWDs were under-challenged by the first set of games. Consequently, additional games were developed to provide new stimuli and variety (see Table 1).

**Table 1.** Games of the KoKoFIT Selection Used in the Different Interventions.

Name (category)	Description		Use in nursing home number
**MusicGame** (cognition)	The “MusicGame” is specially developed for seniors with moderate dementia. Images covered by tiles should be recognized as quickly as possible by removing the tiles. The tiles are removed by moving the cursor using the dislocation of the COG in the respective direction of movement (to the left, right, front or back). The hidden image gradually becomes visible. To encourage the player to keep constantly moving, music plays when the player is actively moving. Images from different categories can be chosen according to the player’s preferences	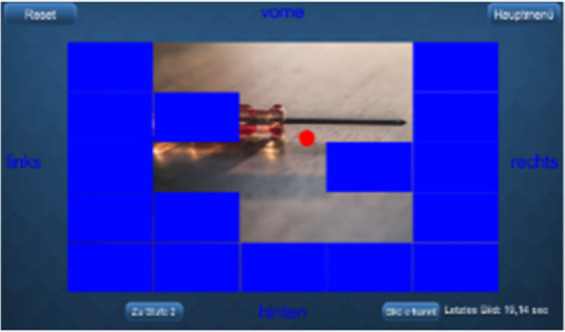	All seven nursing homes
**BalanceBall** (coordination)	In the game “BalanceBall,” a virtual gaming table is tilted so that the silver metal ball moves and the objects on the table are collected. BalanceBall consists of three levels, which should be completed in at least 10 minutes. At the end, a leaderboard shows the ranking of the individual players	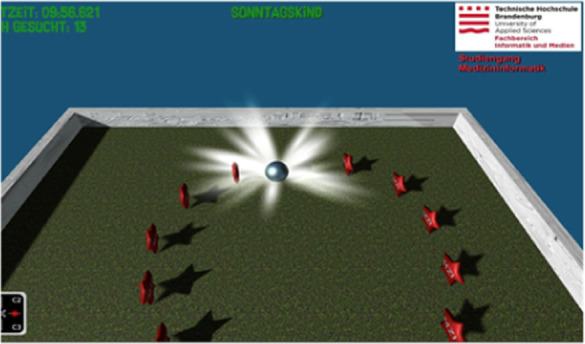	All seven nursing homes
**Forest Walk** (coordination)	In the game “Forest Walk,” a human avatar is controlled, for example, in a realistic-looking forest or on a surfboard steering across the sea. The game aims to collect objects (e.g., pictures of family members) scattered around the forest. For a more realistic experience, typical background noises, for example, birdsong, are audible	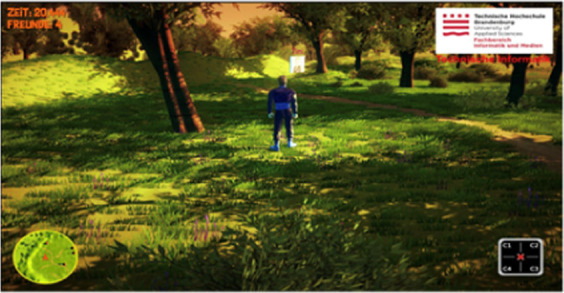	Nursing homes 5 to 7
**PickIT** (cognition)	The “PickIT” game, which is also especially suitable for seniors with moderate dementia, has two game modes: Either a picture or a word is shown, and the appropriate word or picture has to be selected from four words or pictures shown, respectively. The respective words or pictures are selected by shifting the body’s COG.	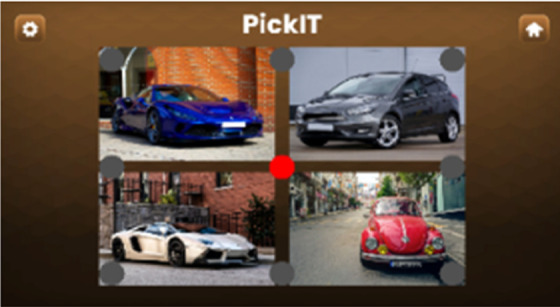	Nursing homes 3 to 7
**Surflex** (coordination)	In the game “Surflex,” objects (beach balls) and thus points are collected. When colliding with obstacles, the player receives minus points. As a cognitive task, numbers/words appear for a short interval on the screen within each level, which the player is supposed to remember and which are queried in the form of a quiz at the end of the round. Using the “Reaction Game,” the reaction speed can be tested. Therefore, the user has to shift the COG toward the flashing arrow as quickly as possible. The average reaction time is recorded	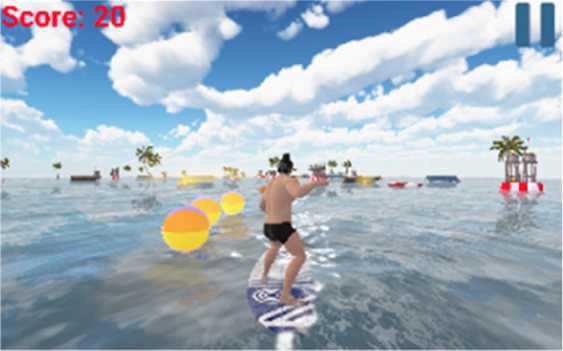	Nursing homes 3 and 5 to 7
**MemoryGame** (cognition)	The “MemoryGame” is the digital version of classic memory, in which pairs of cards must be found from a set of facedown-presented playing cards. Five levels with two to ten pairs of cards can be played consecutively	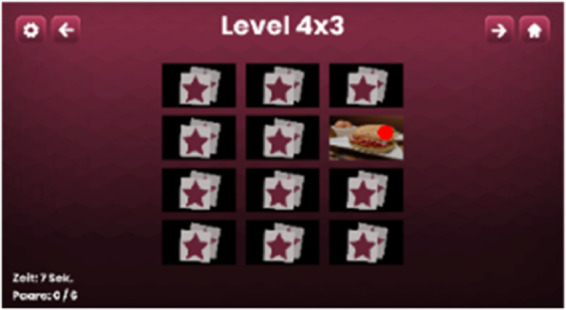	Nursing homes 5 to 7

At the beginning of the study, the test administrator selected the game to assess which games could be performed by the participants and which were not feasible. As the study progressed and the participants’ abilities became clearer, they could choose the games themselves. Only in certain cases (e.g., could not decide) was the game chosen by the test administrator. The game did not have to remain the same for each session; when the test administrator observed that more variety was needed to maintain engagement, the game was changed to provide diversity and sustain participant motivation.

### Instruments and Procedure

Test procedures were used to investigate the study objectives, which, as demonstrated in various studies, can also be carried out on PWD (López-Ortiz et al., 2023). To avoid overburdening PWD with several test procedures, motor tests were carried out one day, and cognitive tests and quality of life were carried out another day.

The MMSE and the Trail-Making-Test A (TMT-A) were used to document the cognitive functions. The MMSE is a test for the general cognitive functioning level used to estimate the degree of dementia. It was completed as an interview and contains questions concerning, for example, orientation, memory, and attention (Folstein et al., 1975). A maximum total score of 30 points could be achieved. To perform the TMT-A, the test subjects connect the numbers 1 to 25 in ascending order (Seo et al., 2006). The time required and a shorter time are associated with better attention. The quality of life (WHOQOL-BREF) and the level of depression (Geriatric Depression Scale) were additionally captured using questionnaires to determine changes in mood and other personal characteristics of the subjects. The WHOQOL-BREF is a 26-item short form of the WHOQOL-100, developed to assess subjective quality of life across four domains: Physical Health, Psychological Health, Social Relationships, and Environment (Skevington et al., 2004). Higher scores indicate better quality of life**.** The Geriatric Depression Scale (GDS-15) is a specially designed questionnaire for detecting depression in older adults. Originally developed by Yesavage et al. (1982), the GDS-15 is tailored to the needs of older adults and excludes age-related physical symptoms not necessarily linked to depression. A low value indicates a lower level of depression.

The functional abilities were examined with static and dynamic balance tests. The static balance tests were conducted on a PLUX force plate (PLUX-Wireless Biosignals S.A, Lisbon, Portugal). The balance of each subject was recorded in four different standing positions: (1) standing on both legs with eyes open and (2) eyes closed, (3) semi-tandem stand with the left or (4) right leg in front for 20 seconds each. The fluctuation’s area was determined; the smaller, the better the balance. Dynamic balance was measured using a dynamic balance test on the force plate. The test subjects had to reach eight points presented on a monitor one after the other by moving or shifting the COG. The eight points then had to be reached as quickly as possible without lifting the heels. Accordingly, the COG had to be moved in four directions (anterior, posterior, left and right). The total time was measured in this digital range test. In addition, the Timed-Up-and-Go Test (TUG) was conducted to assess mobility (Podsiadlo & Richardson, 1991). This test required subjects to stand up from a chair, walk 3 m, make a 180-degree turn, walk back, and sit down again. The time taken by the test subjects was measured. A shorter time required can be associated with better mobility. Regarding functional abilities, the Falls Efficacy Scale-International (FES-I, German version) was additionally used to determine the subjective assessment of fall risk (Hill et al., 1996). During the FES-I, the participants are asked about their fear of falling in different situations (Hill et al., 1996). The score is also calculated based on the answers given, and similarly, higher values are associated with a higher fear of falling (max. 64 points).

Feasibility and acceptability were assessed through participation rates and participant feedback (Feedback form and Observation protocol).

### Data Analysis

The statistical analysis was conducted as a pre-post comparison. Statistical data analysis was performed using SPSS, version 28 (IBM). All quantitative variables were indicated as mean ± standard deviation. The data were analyzed with a Wilcoxon-test. The certain significance values (*p*) were again adjusted with a Dunn–Bonferroni–Holm correction. The significance level was set to α = .05. The effect size was divided into: r = .10 small, r = .25 medium and r = .40 strong effects. Concerning feasibility, the use of the individual games was examined descriptively in terms of frequency and duration.

## Results

### Sample

Twenty-six subjects dropped out during the intervention phase. This corresponds to a drop-out rate of 42.6%. The reasons were cognitive and motor decline (severe functional impairment requiring extensive assistance or dependent on a wheelchair, illness) or hospitalization. Participants were excluded if they had a severe functional impairment requiring extensive assistance, were permanently wheelchair-bound, or were ill or hospitalized for several days, rendering them unable to physically participate in the intervention or to complete at least 75% of the intervention units. The characteristics of the dropouts are presented in Table 2.

**Table 2.** Sample Characteristics.

	Intervention group (*n* = 35)	Drop-out (*n* = 26)
Age (years)		
Mean ± SD	83.17 ± 5.06	82.81 ± 7.34
Sex (%)	m: 8 (22.2 %)	m: 5 (19.2 %)
	f: 27 (77.8 %)	f: 21 (80.8 %)
MMSE score		
Mean ± SD	18.61 ± 5.8	23.19 ± 5.6
Exercise (game) time (min)		
Mean ± SD	12.53 ± 2.42	/
Attendance at practice sessions (%)	89%	/
Degree of dementia		
No dementia (%)	2 (5.7 %)	4 (15.5 %)
Mild dementia (%)	17 (48.7 %)	14 (53.8 %)
Moderate dementia (%)	15 (42.9 %)	7 (26.9 %)
Severe dementia (%)	1 (2.7 %)	1 (3.8 %)

At the end of the study, a total of thirty-five subjects (age: 83.17 ± 5.06 years) were included in the analysis. A post-hoc power analysis was carried out with the 35 subjects (d = 0.5, *p* = .05), and a power of 0.88 was determined.

### Sample Characteristics

Table 2 shows the sample data of the participants. More women participated (77.8%) in the study, and the participants had an overall attendance of 89% in all exercise sessions. The degree of dementia shows that two subjects had no dementia, and one had severe dementia. The data were collected using the MMSE, a standardized cognitive screening test. In this study, the classification of dementia severity is based on MMSE scores. This screening test is sometimes not sensitive enough and may potentially influence the categorization, especially in moderate and severe dementia (Zettl & Sieb, 2021).

### Feasibility

To assess feasibility, both a feedback form and an observation sheet were used to capture participants’ enjoyment and engagement with the games. Analysis of these measures indicated that the participants enjoyed the games, as evidenced by positive verbal expressions such as laughter and comments about the games being entertaining. Additionally, the observation sheet highlighted high levels of active participation and engagement during the sessions, further supporting the interpretation that the games were well-received by the participants. Furthermore, the playing times were analyzed to provide insights not only into the games themselves but also into the frequency with which specific games were played. Due to the recent development of additional games, the individual playing times of only 21 test subjects could be analyzed. The analysis showed that the test subjects accepted the various games, used them in various ways, and enjoyed the experience. Specifically, 31.5% engaged with the Balance-Ball, 14.53% with the Music-Game, 22.59% with PickIT, 16% with Surflex, 6.59% with Forrest Walk, and 8.79% with the Memory game. It was observed that individuals with mild dementia tended to play the more complex games (e.g., Balance-Ball), whereas those with moderate dementia preferred simpler games (e.g., Music-Game, PickIT). All games were accessible and playable by all participants individually.

### Physical Tests (TUG, Balance Tests, FES)

The study results show a significant improvement after the intervention only in the dynamic balance examination (*p* = .013). The participants improved by a median of 8.5 seconds. The other tested parameters, including balance with eyes open and closed, the semi-tandem stance balance standing with the left or right leg in front, the TUG, and the Falls Efficacy Scale International, did not show significant differences between the pre-and post-test results. Descriptively, a slight improvement was observed in the TUG-Test (1 second) and in the FES-I (1.5 points) (see Table 3).

**Table 3.** Pre-post Results of the Motor and Cognitive Test Procedures, as Well as Quality of Life and Depression.

Outcome parameters	Pre-test median (25th/75th percentile)	Post-test median (25th/75th percentile)	Wilcoxon-test (Z-value; *p*-value (Bonferroni-holm))	Effect size r
Motor abilities				
Balance eyes open (mm^2^) 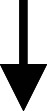	300.00 (240.00/476.00)	360.00 (250.00/644.00)	Z = −0.753 *p* = .451 (>.999)	.13
*n* = 35				
Balance eyes closed (mm^2^) 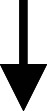	308.00 (235.00/576.50)	376.50 (223.00/615.25)	Z = −1.322 *p* = .186 (>.999)	.22
*n* = 34				
Balance tandem stand left (mm^2^) 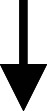	728.00 (416.25/1280.25)	663.00 (408.50/1101.50)	Z = −0.725 *p* = .468 (>.999)	.12
*n* = 32				
Balance tandem stand right (mm^2^) 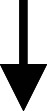	720.00 (448.00/1198.50)	750.00 (588.00/1135.00)	Z = −0.027 *p* = .979 (>.999)	.00
*n* = 33				
Dynamic balance examination (s) 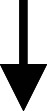	52.00 (42.00/73.50)	43.50 (36.00/51.50)	Z = −3.277 *p* = .001 (.013)	.55
*n* = 29				
Timed-up-and-go-test (s) 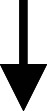	20.00 (14.00/24.00)	19.00 (14.00/23.00)	Z = −0.710 *p* = .478 (>.999)	.12
*n* = 35				
Fall effectiveness scale I (points) 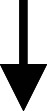	20.50 (17.00/26.50)	19.00 (16.00/22.25)	Z = −1.403 *p* = .161 (>.999)	.24
*n* = 34				
cognitive Abilities				
Mini-mental-state-examination (points) 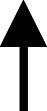	20.50 (15.25/26.00)	22.00 (16.00/26.75)	Z = −1.390 *p* = .165 (>.999)	.23
*n* = 35				
Geriatric depression scale (points) 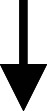	2.00 (1.00/5.00)	2.00 (1.00/4.00)	Z = −0.099 *p* = .921 (>.999)	.02
*n* = 35				
Trail-making-test-A (s) 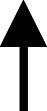	107.00 (70.00/187.00)	115.00 (81.00/158.00)	Z = −0.276 *p* = .782 (>.999)	.05
*n* = 27				
quality of life				
WHOQOL-BREF physical (0-100 scale) 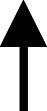	65.50 (55.34/78.56)	67.88 (53.56/76.78)	Z = −0.422 *p* = .673 (>.999)	.07
*n* = 33				
WHOQOL-BREF psychological (0-100 scale) 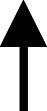	66.69 (62.50/77.08)	66.69 (62.50/72.92)	Z = −0.561 *p* = .575 (>.999)	.09
*n* = 33				
WHOQOL-BREF social relationships (0-100 scale) 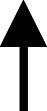	75.00 (64.06/81.25)	65.63 (57.81/75.00)	Z = −1.625 *p* = .104 (>.999)	.27
*n* = 33				
WHOQOL-BREF environment (0-100 scale) 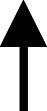	75.00 (66.69/82.73)	67.86 (58.31/75.00)	Z = −2.236 *p* = .025 (.30)	.38
*n* = 33				

Notes. 
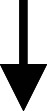
 Lower values are better. 
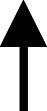
 higher values are better.

### Cognitive Tests (MMST, TMT-A)

The study results show no significant findings in the cognitive parameters. In the MMSE, the median value increased from 20.50 points to 22.00 points, indicating a descriptive improvement, which was not significant (*p* > .999). In the TMT-A, the median value increased from 107.00 seconds to 115.00 seconds, suggesting a slight deterioration, which was also not significant (*p* > .999) (Table 3).

### Additional Tests (WHOQOL-BREF, GDS)

The WHOQOL-BREF domains for physical health, psychological health, social relationships, and environment showed no significant differences between the pre- and post-test results. Similarly, the Geriatric Depression Scale (GDS) remained unchanged. The medians of the pre-and post-test results showed only slight changes, which were not statistically significant (Table 3).

## Discussion

This pilot study explored the feasibility and potential benefits of specially developed serious games for PWD. The primary objective was to determine if these games could be effectively implemented and utilized by PWD. The secondary objective was to investigate whether intervention with these games could positively impact motor skills, cognitive function, quality of life, and levels of depression among the participants. Over a 10-week intervention period, various cognitive and motor functions were assessed pre- and post-intervention.

The results of this study demonstrate that all the games were well-received by PWD and were played with considerable enjoyment. Feedback collected through feedback forms and observation protocols revealed that participants engaged with the games enthusiastically and derived joy from playing. The observations showed that the participants often expressed their enjoyment through positive moods, such as positive verbal comments, and visible expressions, such as smiles and laughter, which further emphasized the attractiveness of the games.

The diverse range of developed games provided various options, ensuring a suitable game for each individual, regardless of their level of dementia. This variety is evident from the playing frequency data, which shows that both simple and complex games were played. Moreover, the data suggests that individuals with mild dementia were more inclined to play complex games like the Balance-Ball. In contrast, those with moderate dementia preferred simpler games like the Music-Game and PickIT. The observation protocols and feedback forms further confirmed that participants consistently found games aligned with their abilities and interests, supporting the usability of the games across varying levels of cognitive impairment. This pattern underscores the importance of providing a range of game complexities to accommodate the varying cognitive levels of PWD.

It is also important to note that all games were accessible and playable by all participants individually. This highlights the games’ usability and adaptability for PWD, ensuring they could engage independently and at their own pace. Furthermore, participants were generally easy to motivate and demonstrated an eagerness to engage with the games. This enthusiasm was evident not only from their verbal and non-verbal feedback but also from the observations made by the study facilitators, who noted the participants’ excitement and satisfaction during gameplay. Collectively, these findings reinforce the effectiveness and enjoyment of the games for PWD.

The results revealed significant improvements in the dynamic balance examination, indicating enhanced balance. However, no significant changes were observed in other motor and cognitive tests, such as the TUG, MMSE, TMT-A, and various quality-of-life measures. However, this can also be seen as a positive result, as PWD usually experience a cognitive and motor decline during this period (López-Ortiz et al., 2023).

The significant improvement in the dynamic balance examination aligns with previous research demonstrating the positive impact of physical activity and balance training on motor function in PWD. Studies by Heyn et al. (2004) and Rolland et al. (2007) have shown that exercise interventions can significantly improve physical and cognitive functions among PWD.

Regarding cognitive abilities, the study showed a descriptive improvement in the MMSE score, but this change was not statistically significant. This suggests that the cognitive impact of the intervention was limited. Studies by Manera et al. (2015) and Anguera et al. (2013) have shown that while serious games can enhance cognitive functions, significant improvements may require longer intervention or additional cognitive training components. The TMT-A results indicated a slight descriptive deterioration, which corresponds to the progressive nature of the dementia. This observation is supported by Abd-Alrazaq et al. (2022), who highlighted the difficulty in achieving significant cognitive gains in PWD through short-term interventions and suggest that longer interventions should be implemented. Notably, there was no major deterioration over time, which is typically expected in PWD (Toulotte et al., 2003).

The quality of life and depression measures, assessed through the WHOQOL-BREF and the Geriatric Depression Scale (GDS-15), did not show significant changes (Ćwirlej-Sozańska et al., 2020). The lack of significant improvement in the WHOQOL-BREF domains suggests that the intervention did not substantially impact the overall quality of life within the study period (Ćwirlej-Sozańska et al., 2020). Similarly, the unchanged GDS-15 scores indicate no significant effect on depression levels. This finding is consistent with other studies suggesting that more intensive or longer-term interventions are required to significantly affect mood and depressive symptoms (López-Ortiz et al., 2023).

The lack of significant findings in other motor and cognitive parameters could be attributed to several factors. Firstly, the short duration of the intervention (10 weeks) may have needed to be increased to observe substantial changes in cognitive functions. The 10-week timeline was primarily determined by logistical and feasibility considerations. Logistically, the availability of facilities, equipment, and staff resources limited the potential for extending the intervention period. Additionally, as this study was designed as a pilot, the shorter duration was chosen to test the feasibility of the intervention within a manageable timeframe, providing valuable insights for future research with extended timelines. Baker et al. (2010) suggest that longer intervention periods are often necessary to detect significant cognitive improvements. Additionally, the heterogeneity of dementia, where motor and cognitive functions decline at different rates among individuals, complicates the assessment of intervention outcomes (Verdi et al., 2021). Several factors could have influenced the study outcomes. One key factor is the individual variability among PWD. Dementia affects each person differently, with some experiencing more pronounced motor deficits while others face significant cognitive declines (Verdi et al., 2021). This individual variability can lead to mixed results in studies that do not tailor interventions to specific patient needs. The one-size-fits-all approach of the games might not have adequately addressed the diverse challenges the participants faced.

Another possible reason for the limited significant findings could be related to the nature of the serious games used. While serious games have shown the potential to enhance cognitive functions and provide engaging activities for PWD (Anguera et al., 2013; Manera et al., 2015), these games’ specific design and implementation are crucial. Manera et al. (2017) highlighted the need for detailed guidelines and standardized protocols for serious games to ensure their effectiveness. While user-centered and developed with input from older adults, the games used in this study might still require further refinement to maximize their impact on cognitive and motor functions.

Moreover, the intensity and frequency of the game-based interventions might have played a role (Vázquez et al., 2018). While the participants engaged in the serious games regularly, the overall intensity of the intervention may not have been sufficient to produce significant cognitive benefits. Despite being engaging and interactive, the games used in this study may need to be integrated with more rigorous and frequent training sessions to achieve desired cognitive outcomes.

Another consideration is the participants’ initial familiarity and comfort with technology. Older adults, particularly PWD, may have varying levels of comfort and experience with digital interfaces and game-based interventions (Neal et al., 2021). Those less familiar with such technology might have faced initial challenges in engaging fully with the games, potentially affecting their performance and the intervention’s overall effectiveness (Neal et al., 2021). Future studies should consider incorporating introductory sessions to acclimate participants to the technology and ensure they can engage fully with the game-based interventions (Ipakchian Askari et al., 2024).

The study also underscores the importance of individualized interventions. Given the variability in how dementia affects motor and cognitive functions, personalized game designs that cater to individual capabilities and progression rates are crucial (Lourida et al., 2020). Tailoring the interventions to address specific needs can help achieve better outcomes and make the interventions more effective (Lourida et al., 2020). The findings suggest that while serious games hold promise, they must be carefully designed and implemented to maximize their impact (Lourida et al., 2020).

From a practical standpoint, the study highlights the potential for serious games to be integrated into dementia care programs with proper adaptation and development. These games can provide an engaging and less resource-intensive alternative to traditional exercise programs, offering a scalable solution to improve the well-being of PWD. By leveraging technology, healthcare providers can create personalized and adaptable interventions that enhance cognitive and physical functions, ultimately improving the quality of life for PWD (Manera et al., 2017).

### Generalizability and Limitations

The generalizability of this study’s findings is limited by its small sample size and short intervention duration. Additionally, the study’s single-group design needs a control group to allow the ability to draw definitive conclusions about the effectiveness of the intervention. To improve the generalizability and robustness of future studies, larger sample sizes and randomized controlled trials are necessary. These studies should also consider longer intervention periods to capture more substantial changes in cognitive and motor functions. In addition, several quantitative values or parameters should be employed to assess feasibility or practicality, enhancing the overall evaluative power regarding feasibility. To obtain accurate information about the degree of dementia, neurologists would have to be consulted, or the Global Deterioration Scale could be used. This study also highlights the notably high dropout rate, limiting the findings.

Future studies should offer more games from the outset to minimize under- or overchallenging and potentially resulting motivation problems and thus create more variety and incentive for the participants.

Moreover, the absence of a follow-up assessment in this study represents a critical limitation. Follow-up evaluations are essential for determining the lasting effects of serious games and assessing whether PWD continues to benefit from the interventions over time. Long-term follow-up studies can provide valuable insights into the sustainability of cognitive and motor improvements and the overall impact on quality of life for participants (López-Ortiz et al., 2023). Furthermore, future research should address the need for standardized protocols and detailed guidelines for designing and implementing serious games for PWD. Establishing consistent methodologies and outcome measures will enhance the comparability of studies and provide more reliable evidence for the effectiveness of these interventions.

## Conclusion

In summary, this pilot study demonstrated that serious games could feasibly be implemented for PWD and showed the potential to improve the dynamic balance. The developed games successfully catered to the diverse needs of PWD, providing both entertainment and cognitive engagement. The variety of games available allowed for individualized game choices, reflecting the different levels of cognitive abilities among the participants. However, the study also highlighted the need for longer intervention periods, more individualized game designs, and larger sample sizes to fully assess the benefits of serious games on cognitive functions and overall quality of life. The study’s results suggest that serious games can play a valuable role in dementia care, particularly in improving motor functions such as balance. However, these games must be carefully designed, individualized, and integrated into comprehensive care programs to maximize their potential.

## Data Availability

The datasets used and/or analyzed during the current study are available from the corresponding author upon reasonable request.*
